# Developing a clinical practice guideline implementation strategy based on needs, evidence and theory

**DOI:** 10.1186/1472-6963-14-S2-P85

**Published:** 2014-07-07

**Authors:** Chirk Jenn Ng, Ee Ming Khoo, Ping Yein Lee, Su May Liew, Stalia Siew Lee Wong, Adina Abdullah, Nik Sherina Hanafi, Yook Chin Chia, Nurdiana Abdullah

**Affiliations:** 1 Department of Primary Care Medicine, University of Malaya, Kuala Lumpur, Malaysia; 2 Family Medicine Department, Serdang, Malaysia; 3 Klinik Perubatan Keluarga SL Wong, Klang, Malaysia

## Background

Clinical practice guidelines (CPGs) are used to standardize care according to evidence-based recommendations. However, the implementation of CPGs in the real world varies; a possible reason for this is the lack of a systematic approach to the implementation of CPG. We, therefore, aimed to develop an intervention to improve the implementation of a local hypertension CPG based on the needs of healthcare professionals (HCPs), best available evidence, and theories.

## Materials and methods

This study was conducted in 2013 at an urban hospital-based primary care clinic in Malaysia which used a paper-based medical record system. The 2008 national CPG on hypertension was used for the research. The intervention was developed based on: (1) findings from an audit and needs assessment study involving the PCPs, pharmacists, nurses and the administrators; (2) literature review of the effectiveness of various CPG implementation strategies in primary care; and (3) the theory of planned behaviour.

The research team convened to summarise the key findings from the needs assessment study and reached a consensus on two major issues: lack of accessibility to the CPG and collaboration among the HCPs. The literature review highlighted the need for a multi-faceted strategy which should include dissemination and implementation.

## Results

A need-, evidence- and theory-based intervention was developed after a few iterations and feedback from the users. It comprised several components. Firstly, two training sessions were conducted by two senior academicians to increase the PCPs’ knowledge on the assessments and treatment of hypertension based on the CPG. Secondly, a quick reference guide summarising key recommendations from the CPG was placed on table tops as well as on the computer screen of each consultation room for easy access and reference. Thirdly, prior to the consultation with the doctor, the patient completed a self-assessment form and this was followed by the measurement of anthropometric parameters by the nurses. Requisition forms for investigations recommended by the CPG were placed together with patient medical records for ease of access and to facilitate doctors in making a treatment decision. Finally, a personalized checklist was placed in the patient medical record to serve as a quick reference and reminder to the doctor and it included items such as cardiovascular risk assessment.

## Conclusions

This study showed that it is feasible to develop a CPG implementation strategy based on needs, evidence and theories. We are in the process of evaluating the intervention.

